# Supplementary material for: Comprehensive Gene Expression Analyses of Immunohistochemically Defined Subgroups of Muscle-Invasive Urinary Bladder Urothelial Carcinoma
Source: Int J Mol Sci. 2021 Jan 10;22(2):628. doi: 10.3390/ijms22020628 (PMC7828072; doi:10.3390/ijms22020628)
Supplement: Supplementary file 1 [file ijms-22-00628-s001.zip › UCC_vIJMS_supple1.pdf]

International Journal of
Molecular Sciences

MDPI

(a)

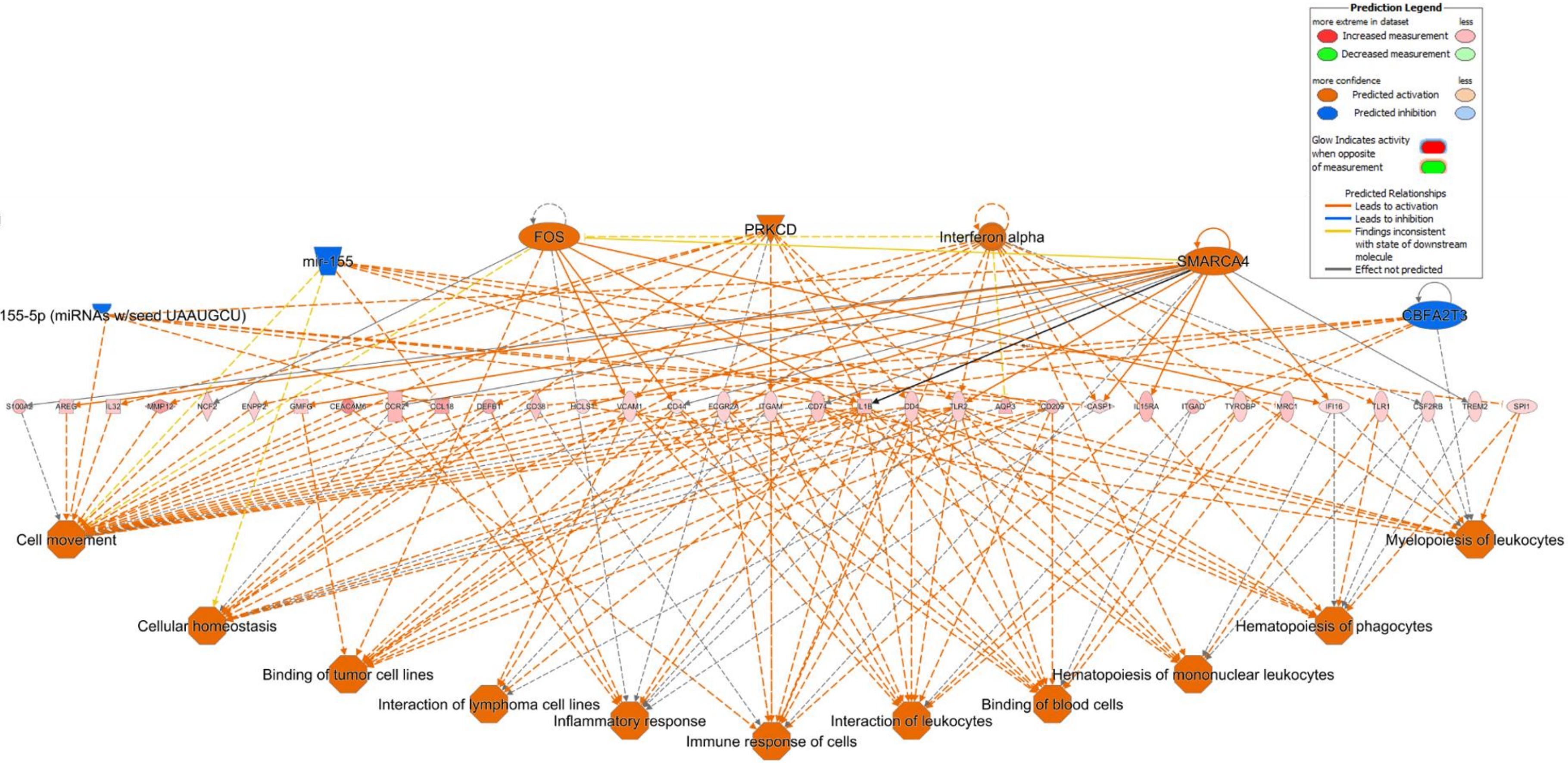

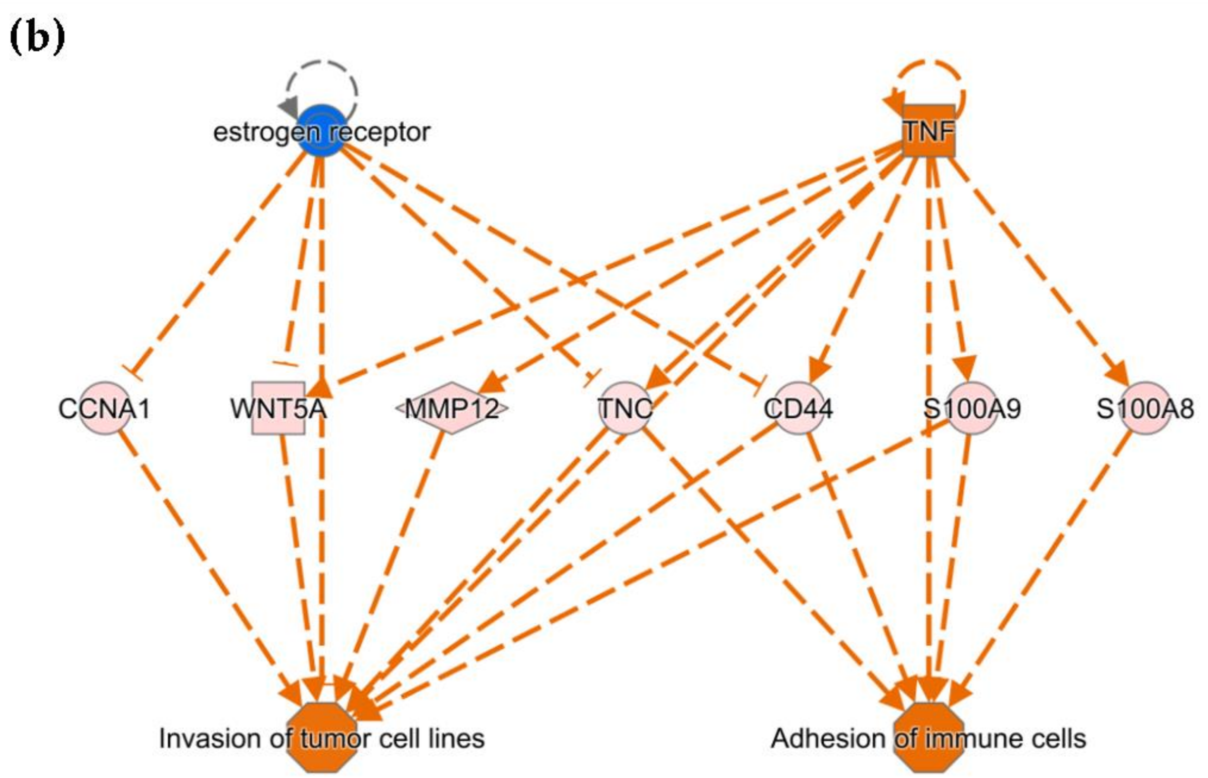
(b)
estrogen receptor
TNF
CCNA1
WNT5A
MMP12
TNC
CD44
S100A9
S100A8
Invasion of tumor cell lines
Adhesion of immune cells

(c)

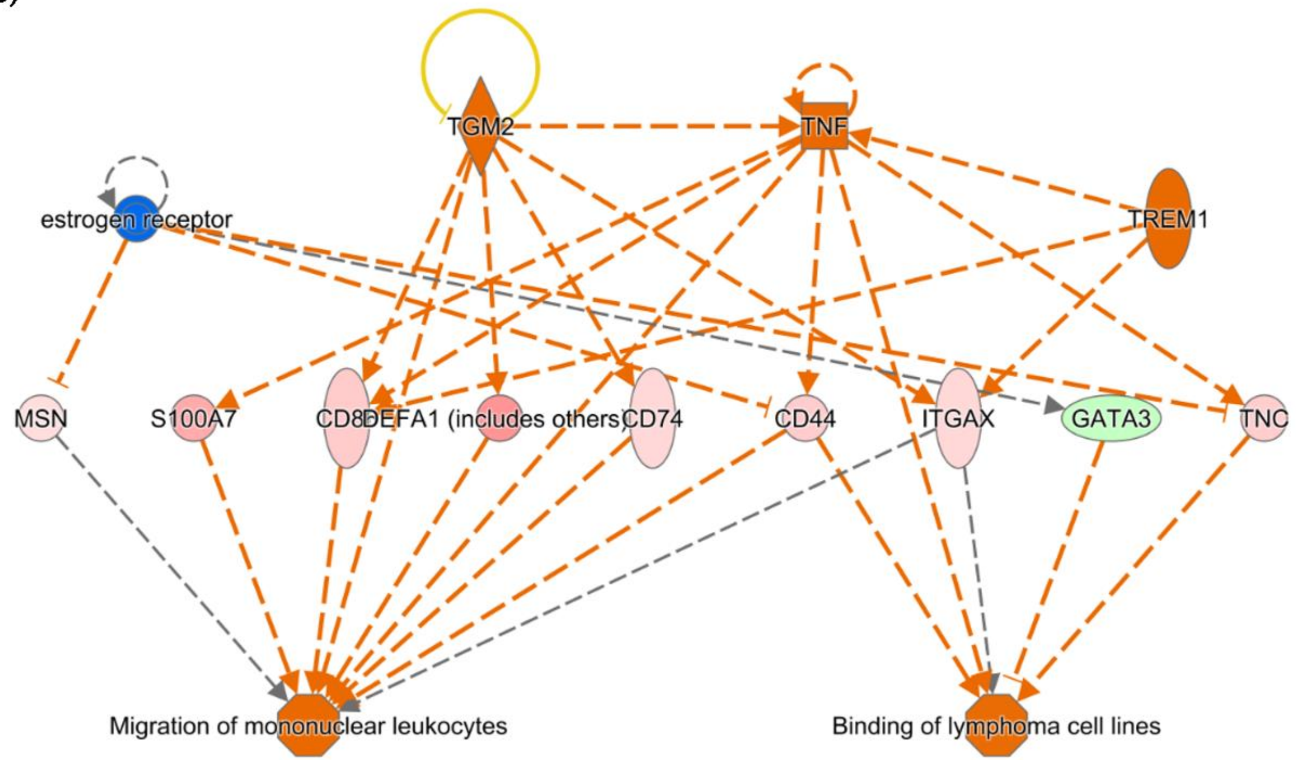
TGM2
TNF
estrogen receptor
TREM1
MSN
S100A7
CD8
DEFA1 (includes others)
CD74
CD44
ITGAX
GATA3
TNC
Migration of mononuclear leukocytes
Binding of lymphoma cell lines

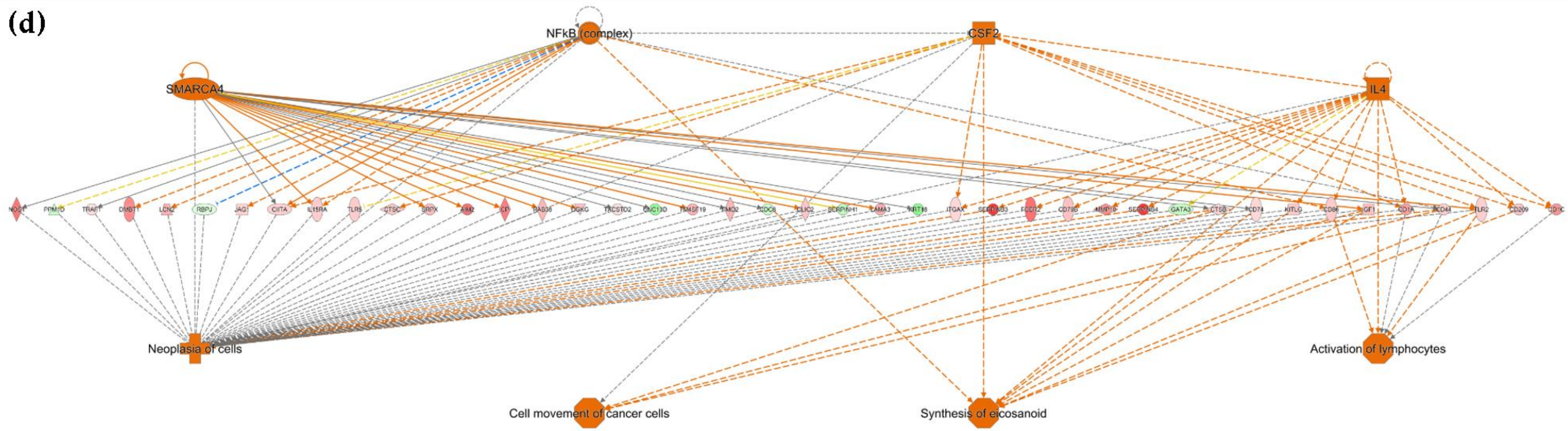

Figure S1. The Ingenuity Pathways Analysis results; Regulator effect between four subgroups: (**a**) DP vs DN; (**b**) CK5/6 SP vs CK20 SP; **(c)** CK5/6 SP vs DN; **(d)** CK5/6 SP vs DN.
