# Supplementary material for: Comprehensive Gene Expression Analyses of Immunohistochemically Defined Subgroups of Muscle-Invasive Urinary Bladder Urothelial Carcinoma
Source: Int J Mol Sci. 2021 Jan 10;22(2):628. doi: 10.3390/ijms22020628 (PMC7828072; doi:10.3390/ijms22020628)
Supplement: Supplementary file 1 [file ijms-22-00628-s001.zip › UCC_vIJMS_supple2.pdf]

International Journal of
Molecular Sciences

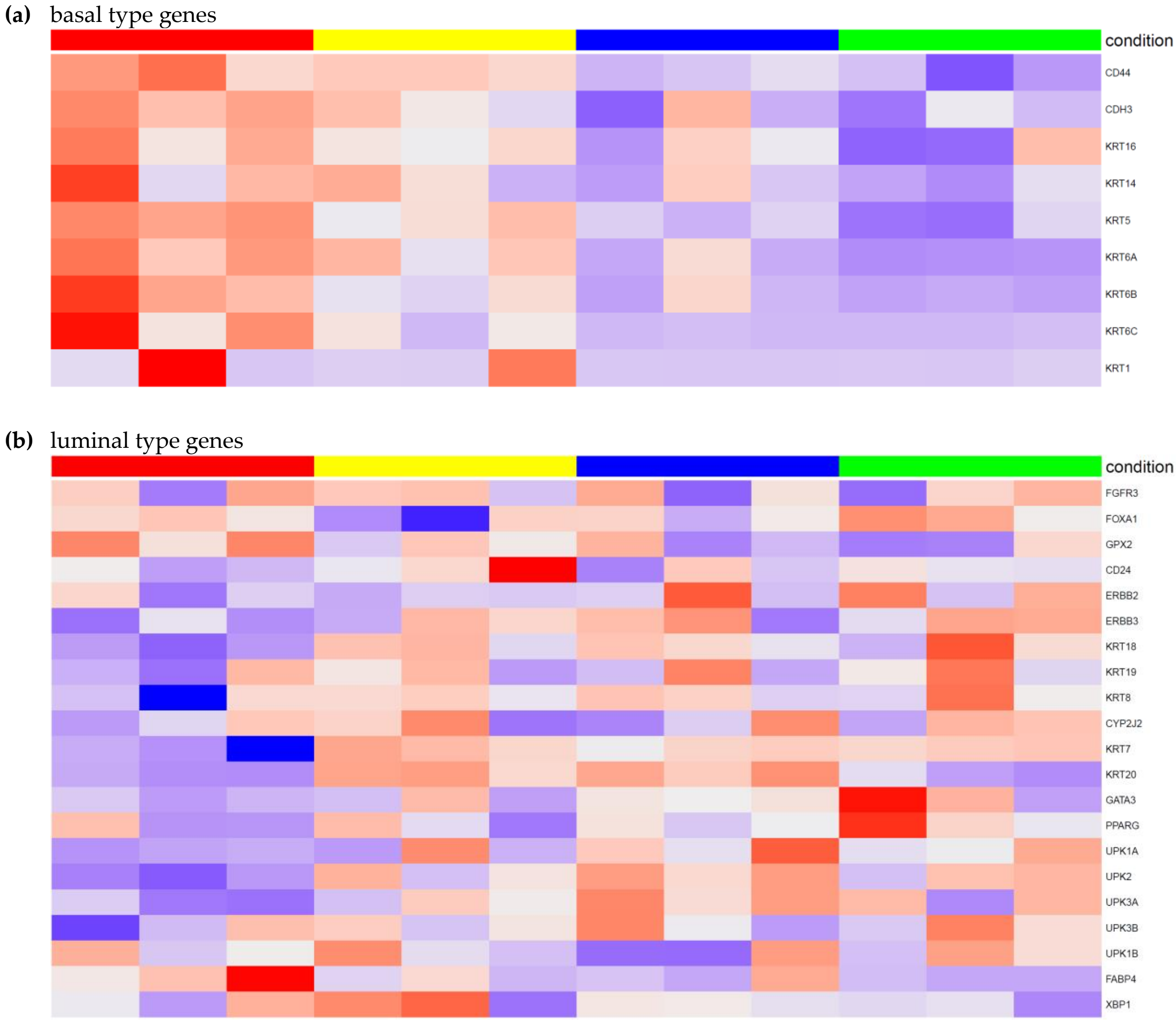

(c) p63-associated genes

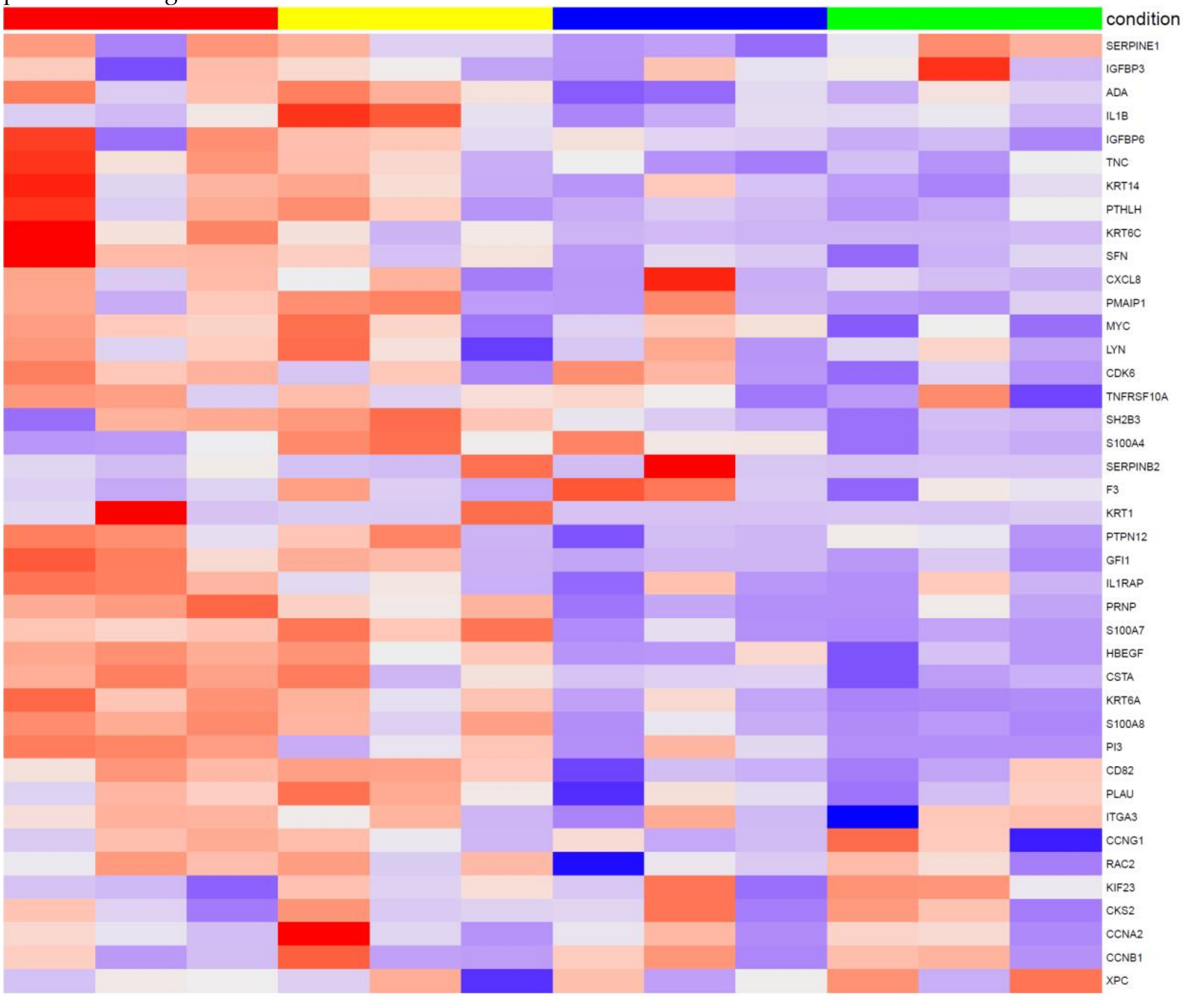

(d) TP53-like signature genes

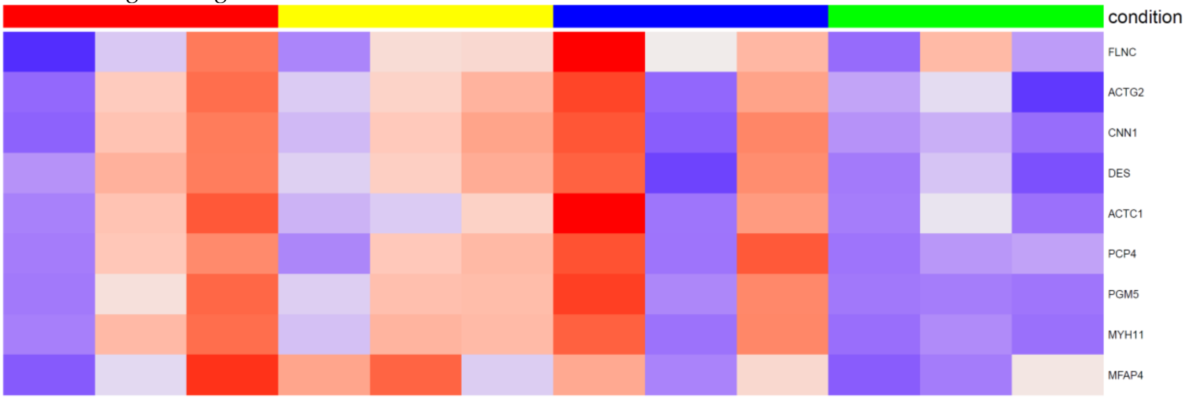

(e) immune response genes

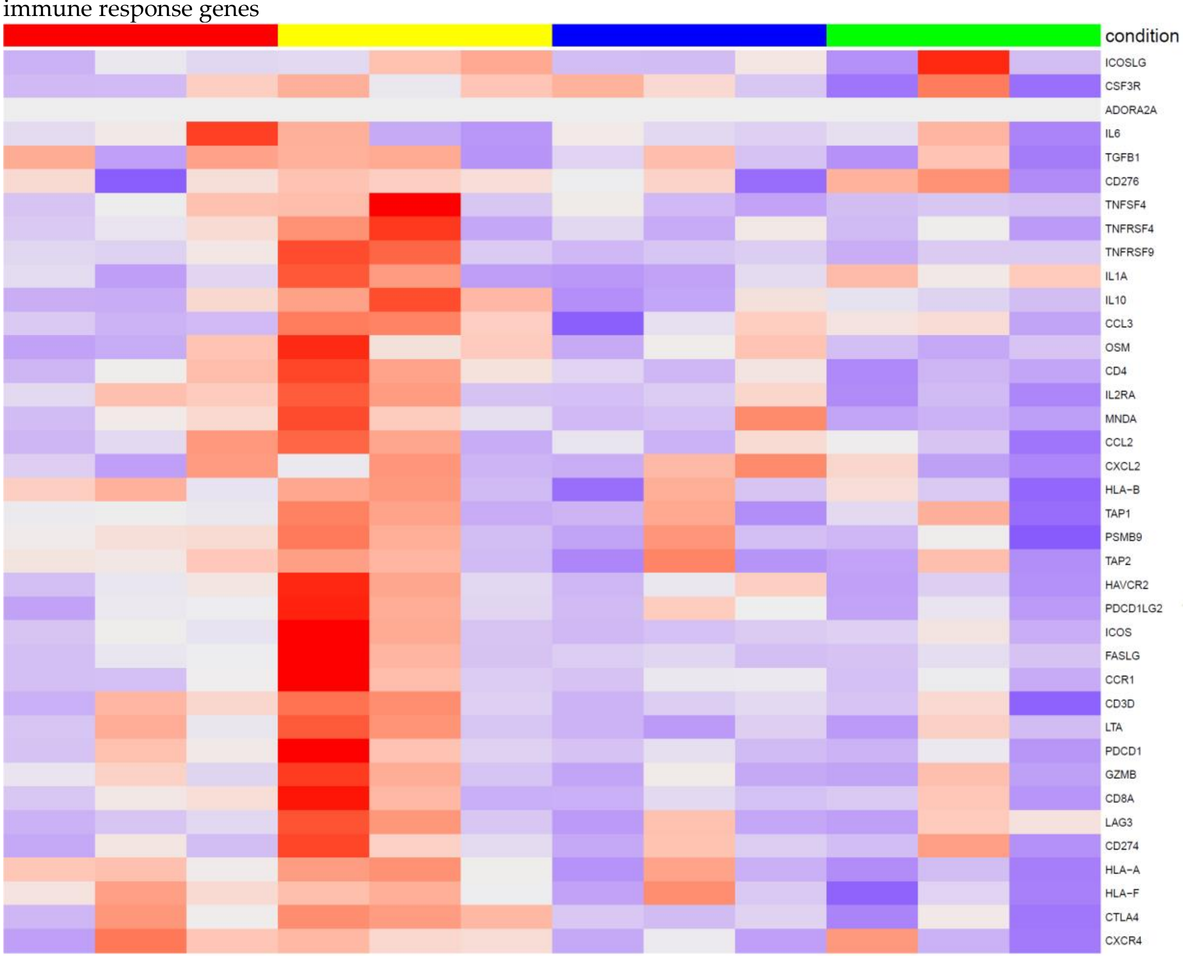

(f) epithelial-mesenchymal transition genes

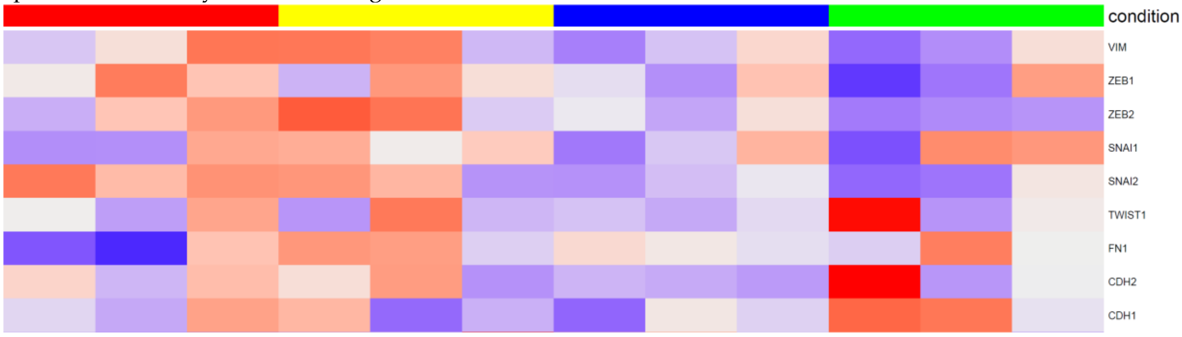

(g) cell adhesion genes

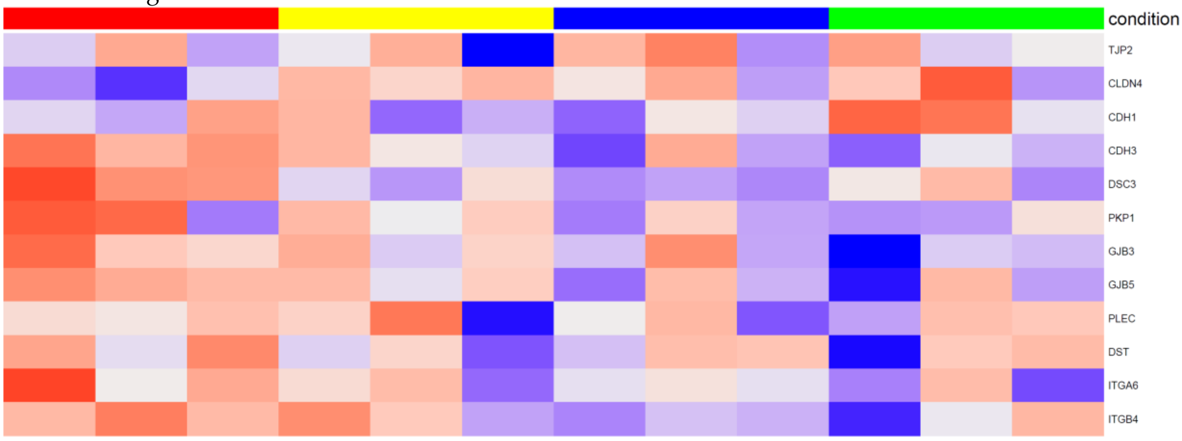

(h) MAPK signaling pathway genes

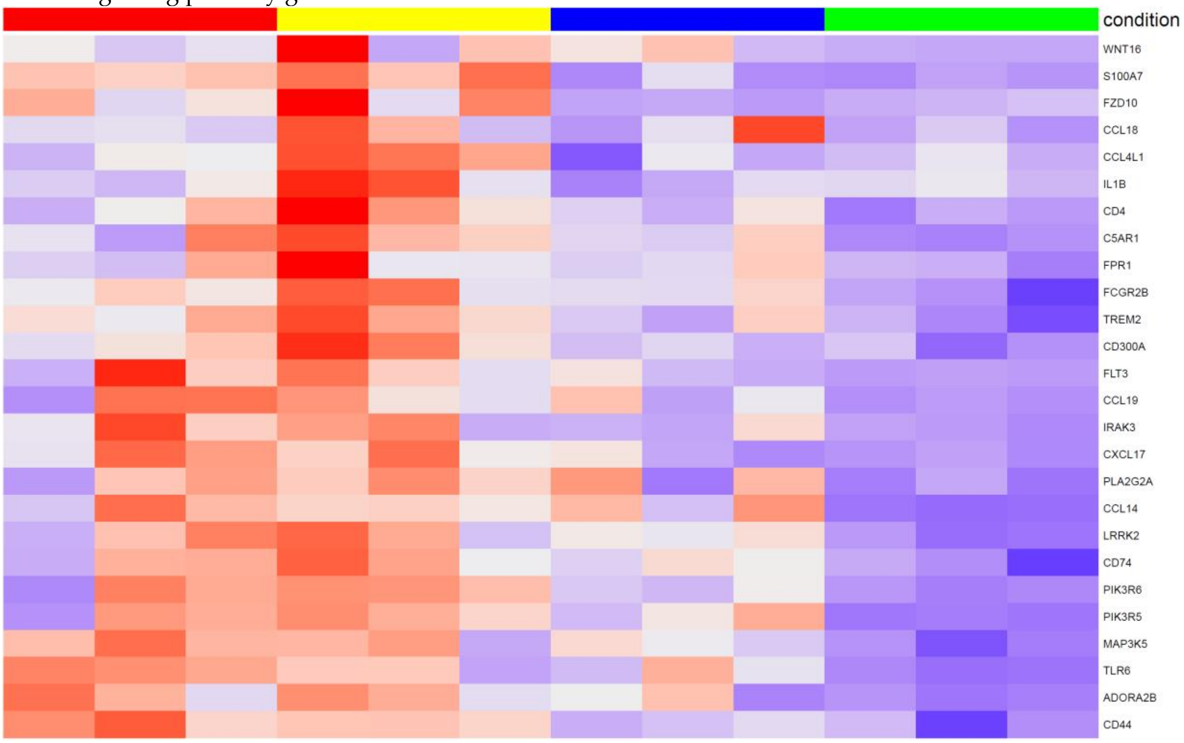

(i) TNF signaling pathway genes

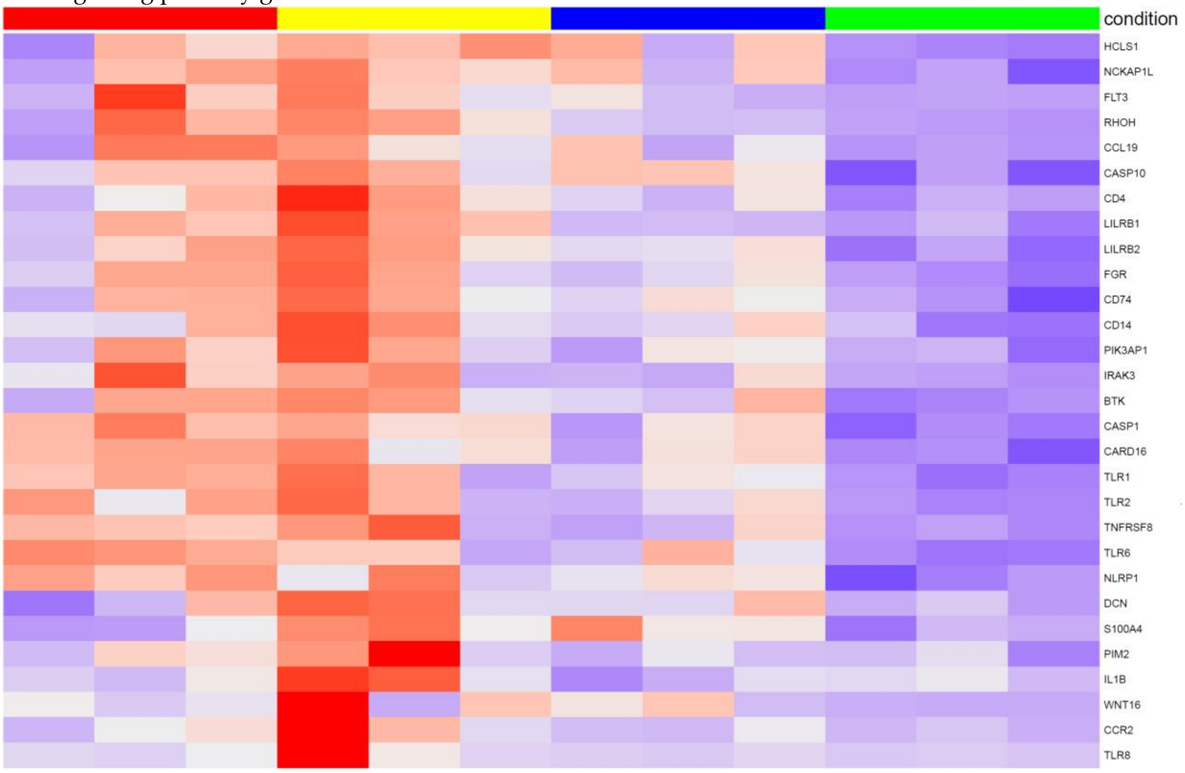

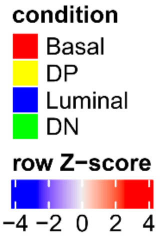

Figure S2. Expression of gene expression signature between four subgroups: (**a**) basal type genes; (**b**) luminal type genes ; **(c)** p63-associated genes; **(d)** TP53-like signature genes; **(e)** immune response genes; **(f)** epithelial-mesenchymal transition genes; **(g)** cell adhesion genes; **(h)** MAPK signaling pathway genes; **(i)** TNF signaling pathway genes.
